# Development of HA/Ag-NPs Composite Coating from Green Process for Hip Applications

**DOI:** 10.3390/molecules22081291

**Published:** 2017-08-08

**Authors:** Denisse A. Lozoya-Rodríguez, Renata de Lima, Leonardo F. Fraceto, Antonio Ledezma Pérez, Mercedes Bazaldua Domínguez, Roberto Gómez Batres, Armando Reyes Rojas, Víctor Orozco Carmona

**Affiliations:** 1 Centro de Investigación en Materiales Avanzados (CIMAV), Miguel de Cervantes 120, Chihuahua 31109, Mexico; denisse.lozoya@cimav.edu.mx (D.A.L.-R.); roberto.gomez@cimav.edu.mx (R.G.B.); armando.reyes@cimav.edu.mx (A.R.R.); 2 Departamento de Biotecnologia, Universidade de Sorocaba (UNISO), Rod. Raposo Tavares, km 92.5, Sorocaba 18023-000, Brazil; renata.lima.ppba@gmail.com; 3 Departamento de Engenharia Ambiental, Universidade Estadual Paulista (UNESP), Av. Três de Março 511, Sorocaba 18087-180, Brazil; leonardo@sorocaba.unesp.br; 4 Centro de Investigación en Química Aplicada (CIQA), Blvd. Enrique Reyna 140, Saltillo 25250, Mexico; antonio.ledezma@ciqa.edu.mx; 5 Centro de Capacitación, Investigación, Innovación y Transferencia Tecnológica, Universidad Tecnológica de Chihuahua Sur (UTCh Sur), Km. 3 Carretera Chihuahua-Aldama, Chihuahua 31313, Mexico; mbazaldua@utchsur.edu.mx

**Keywords:** biological hydroxyapatite, coating, silver nanoparticles, atmospheric plasma spray, antibacterial test, cytotoxic test

## Abstract

In the present study, biological hydroxyapatite (HA) was obtained from bovine bones through a thermal process. A total of 0% and 1% of silver nanoparticles (Ag-NPs) synthesized from *Opuntia ficus* (nopal) were added to the biological hydroxyapatite coatings using an atmospheric plasma spray (APS) on a Ti6Al4V substrate. Following this, its antimicrobial efficiency was evaluated against the following bacterial strains: *Escherichia coli*, *Staphylococcus aureus*, and *Pseudomonas aeruginosa*. This was conducted according to the Japanese Industrial Standard (JIS) Z2801:2000 “Antimicrobial Product-Test for Antimicrobial Activity and Efficacy”. Scanning electron microscopy (SEM) showed that the silver nanoparticles (Ag-NPs) were evenly distributed on the coating surface. Energy dispersive X-ray spectroscopy (EDX) shows that apatite deposition occurs on a daily basis, maintaining a Ca/P rate between 2.12 and 1.45. Biocompatibility properties were evaluated with osteoblast-like cells (MC3T3-E1) by single-cell gel electrophoresis assay and Tali image cytometry.

## 1. Introduction

Calcium phosphate salts (CaP) are the major mineral component of bones and teeth in vertebrates. Bones and other calcified tissues can be considered as natural anisotropic composites of biomaterials that are embedded in a protein matrix, other organic matter, and water. Bones contain approximately 65–70% of biomineral phase (composed of one or more types of calcium phosphates), with water constituting 5–8%, and the rest corresponding to the organic phase, which is mostly collagen. Hydroxyapatite (HA) is the most thermodynamic stable calcium phosphate salt inside the fluids of the human body. Likewise, it has the greatest similarity to the mineral part of the bone. HA is always found as a carbonated compound in bones and other calcified tissues, and is thus calcium deficient. As a result, its Ca/P rate is less than 1.67 [1,2,3]. HA has been used in coatings to promote the bonding of prostheses through osseointegration [4].

It has been reported that infections related to implants play an important role as one of the most common clinical complications. The post-operation infection rate is 5% in primary cases, 6% for revision cases, and 43% for revision cases of recurrent infections [5]. The pathogenesis of the infection is related to microorganisms that grow in a biofilm, making it difficult to treat these microorganisms [6]. In order to reduce the risk of infection, proper prophylaxis is advised. Due to the limited blood irrigation present in bone tissue, there is subsequently limited distribution of antibiotics in infected areas. In order to reduce the rate of infection, the concentration of antibiotics is increased. However, higher concentrations of antibiotics in blood for a long duration result in antibiotic toxicity. There exist both organic and inorganic antibiotics. Organic antibiotics form a biofilm on the implant surface, increasing the bacterial resistance to antibiotics. In comparison, inorganic antibiotics are antimicrobial agents that do not induce bacteria resistance, and are thus an excellent option for local antimicrobial treatment [5].

The development of coatings with excellent antimicrobial activity and good osseointegration is essential for preventing costly therapies or implant removal [6]. Such coating materials can be applied using different methods, such as sputtering, ionic exchange, sol-gel, plasma spray, etc.

It is a well-known fact that silver and silver-based compounds are highly toxic to various microorganisms of clinical importance. Furthermore, when silver nanoparticles are used, there is a dramatic increase in the surface area available, resulting in greater exposure for the microorganism. The exact mechanism for the antimicrobial effects of silver nanoparticles is not clearly known and is a debated topic, although there are various theories proposed about their actions [7,8]. These theories describe the ability of silver nanoparticles to anchor to the bacterial cell wall and generate structural changes, such as increasing the permeability of the cell membrane, which eventually causes cell death. Furthermore, the formation of free radicals by silver nanoparticles is thought to play an important role. These free radicals have the ability to damage the cell membrane and make it porous, which can ultimately lead to cell death. It has also been proposed that there are silver ions released from the nanoparticles, which interact with the thiol groups of many vital enzymes and inactivate them. Another case is the generation of reactive oxygen species, which are produced through the inhibition of a respiratory enzyme by silver ions and attack the cell itself. Nanoparticles also act on sulfur and phosphorus, which are major components of DNA. This destroys the DNA, which would definitely lead to cell death.

Many techniques of synthesizing silver nanoparticles by a variety of chemical and physical methods have been reported in the literature. Unfortunately, these techniques are relatively expensive and also involve the use of toxic, hazardous chemicals, which may pose potential environmental and biological risks. However, the techniques for the synthesis of silver nanoparticles via a green route using biological molecules and plant extracts could be an alternative to conventional methods. The greener synthesis of silver nanoparticles using plant extracts is also more advantageous than other methods as it is environment friendly, easily scaled up, relatively reproducible, and provides natural capping agents for nanoparticle stabilization. Furthermore, there is no need for the use of high temperature, pressure, energy, or toxic chemicals [9,10].

Plant extracts may act both as reducing agents and stabilizing agents in the synthesis of nanoparticles due to the different biomolecules (phenolic compounds, oligosaccharides, free sugars, proteins, and so on) present in their composition. Some Fourier transform infrared spectroscopy (FTIR) studies have confirmed the fact that the amide group from proteins present in the extracts has a stronger ability to bind metal, which indicates that proteins could possibly form a layer covering the metal nanoparticles (i.e., capping of silver nanoparticles) to prevent agglomeration. Through this ability, proteins would stabilize and protect the oxidation [10,11].

Studies have been developed to improve the antibacterial properties of HA coating by the addition of silver nanoparticles (Ag-NPs) [12]. In previous studies, we developed HA and Ag-NPs coating through atmospheric plasma spray (APS) using chemically processed components [13].

The science of biomaterials involves the study of physical, biological, and chemical characteristics of materials, as well as the respective evaluations and interactions between the material and the receptive body. Biocompatibility is the ability of a material to perform under an appropriate host response in a specific application [14]. The biocompatibility of a medical device covers both compatibility and design [15].

Currently engineered biomaterials are able to be in contact with living tissue during extended periods of time as part of the tissue to target the improvement of functionality or tissue completion [16]. One of the most important characteristics of implant materials is the presence of porosity or cavities, which allow tissue to grow toward the material in order to improve bonding [4].

In recent years, researchers have been developing HA synthesis, including employing food waste as precursor, or the extraction of HA from animal bones [1]. There are certain differences between synthetic HA and natural or biological HA. The latter has better metabolic activity and a higher dynamic response to the environment, making biological HA a more ideal implant coating material [17]. One of the reasons for this behavior is that biological HA is non-stoichiometric due to the trace amount of ions incorporated in its crystal structure, such as Fe^2+^, Mg^2+^, Si^2+^, and F^−^ [18].

There are some inorganic antibacterial agents that can be integrated in biomaterial coatings, such as silver and its ions. Silver is well known for its effective inhibitory effects on a wide spectrum of bacteria [5]. On the other hand, the use of silver is limited due to the toxicity of its ions. However, nanotechnology has extended the possibilities of its use with particles of large superficial areas and small sizes, which improves its efficacy and reduces its toxicity [19]. Still, the mechanism of cytotoxicity induction is not well understood. It has been suggested that Ag-NPs act like Trojan horses, finding their way inside the cell and releasing silver ions that impair intracellular functions. However, Kim et al. argued that the cytotoxicity of Ag-NPs is the result of oxidative stress and is completely unrelated to the toxicity of silver ions [20]. A better understanding of this issue is crucial for the cytotoxic evaluations of Ag-NPs [12]. The use of non-toxic and environmentally friendly synthesis methods for nanoparticles is very attractive, especially if they are intended to be used in invasive applications in the medical field. To achieve this, microorganisms, plant and fruit tissue, living plants, extracts of plants, and some marine algae have been used. This biogenic synthesis is useful due to its low environmental impact and production of nanoparticles that are free of chemical traces [21].

## 2. Results

**Figure 1:**
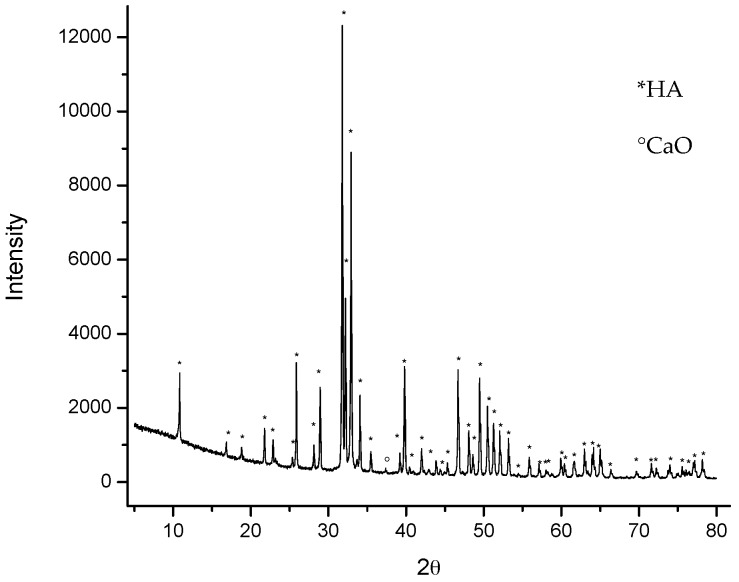
X-ray power diffraction (XDR) pattern of Hydroxyapatite (HA) obtained from bovine bones at 800 °C.

The X-ray power diffraction (XDR) analysis (Figure 1) shows that the material produced after the thermal process is HA, according to the 00-074-0565 diffraction pattern standard from the International Centre for Diffraction Data (ICDD). The pattern also reveals a minor presence of CaO, indexed with the ICDD card 00-075-026.

**Figure 2:**
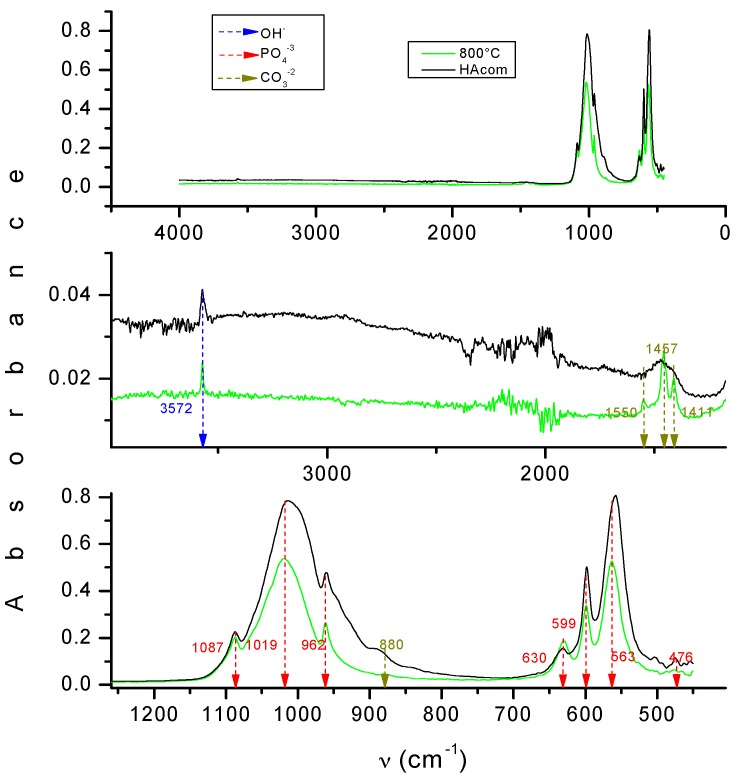
Fourier transform infrared spectroscopy (FTIR) spectra of HA obtained from bovine bones at 800 °C.

The Fourier transform infrared spectroscopy (FTIR) spectrum from the powder is presented in Figure 2, which exhibits the OH^−^, PO_4_^−3^ and CO_3_^−2^ peaks. This result shows that the HA is carbonated. Therefore, it is less stoichiometric and more similar to the composition of real bone.

According to the chemical formula, the calcium to phosphorous molar ratio (Ca/P) was approximately 1.67. However, because this HA was obtained from bovine bones, the molar ratio was slightly less than the stoichiometric ratio, due to the presence of CO_3_^−2^ and other ions. This was demonstrated with a powders analysis by inductively coupled plasma optical emission spectrometry (ICP-OES), which showed that the molar ratio Ca/P was 1.65. The proportion of each element present in the powder was also analyzed, and it was found that the powder was composed of: 0.049% K, 0.534% Mg, 0.821% Na, and 0.013% Si. The presence of these ions is important since they have a biological role, according to previous studies [1,22,23,24].

The powder had a particle size distribution (PSD) average of 119.75 μm, which is a satisfactory particle size for application in an APS process. Therefore, HA/Ag-NPs coatings were applied successfully.

**Figure 3:**
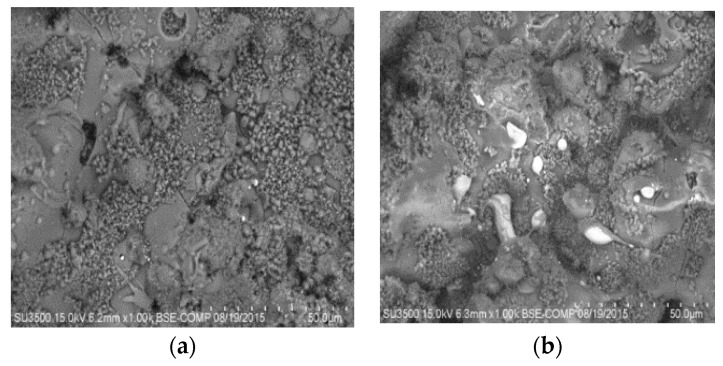
Scanning electron microscopy (SEM) micrographs of the HA/Ag-NPs coatings showing silver. (**a**) zone 1; (**b**) zone 2.

**Figure 4:**
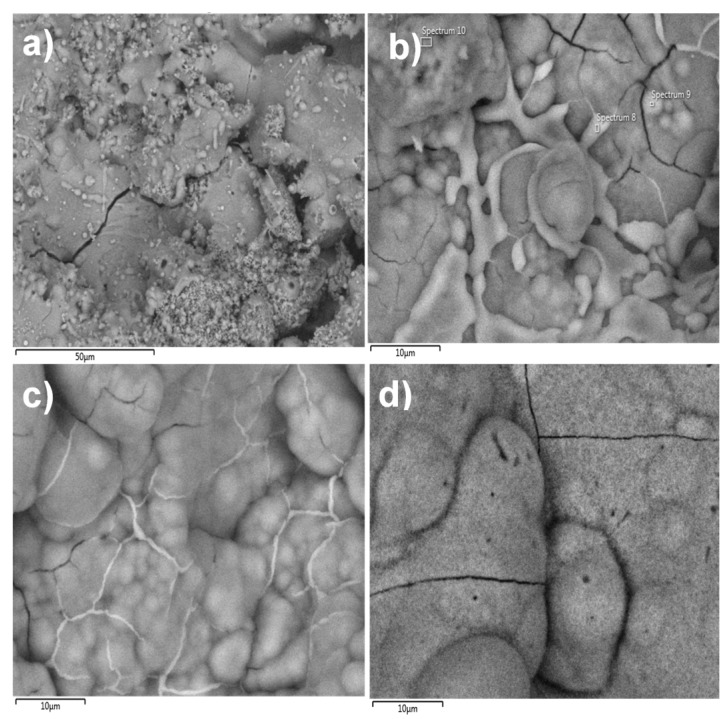
SEM micrographs of the HA/Ag-NPs coating exposed to simulated body fluid (SBF): (**a**) 1 h; (**b**) 12 h; (**c**) 24 h and (**d**) 30 days.

The surface morphology of the coating was observed by scanning electron microscopy (SEM). Figure 3 shows that the coating had good texture, micro-fractures, and good dispersion of silver nanoparticles, even when the powder exhibits an agglomeration tendency. To determine the osseointegration properties, the coatings were submerged in simulated body fluid (SBF) for different periods of time (1, 3, 12 and 24 h as well as 3, 7, 14 and 30 days) and analyzed by SEM. The results showed that apatite was formed on the coating and there was a higher concentration of apatite formation in fractured areas (Figure 4). According to Hatzistavrou et al., the rapid growth of the apatite on the surface of the samples can be associated with the presence of the CaO phase in the natural HA [25].

**Figure 5:**
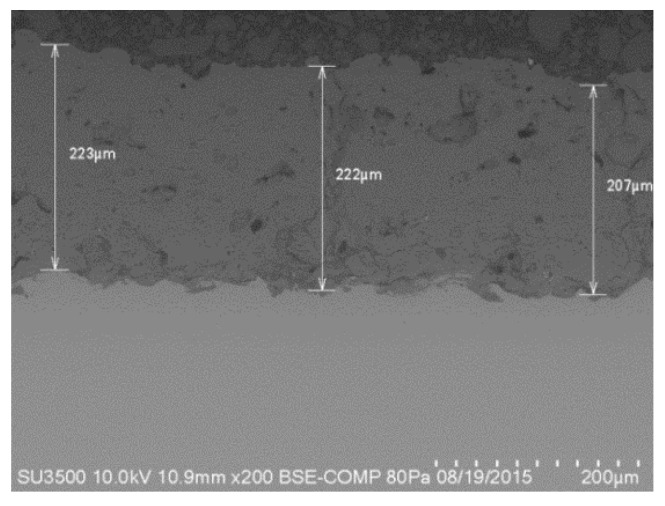
Transversal section from SEM showing thickness of the coating.

**Figure 6:**
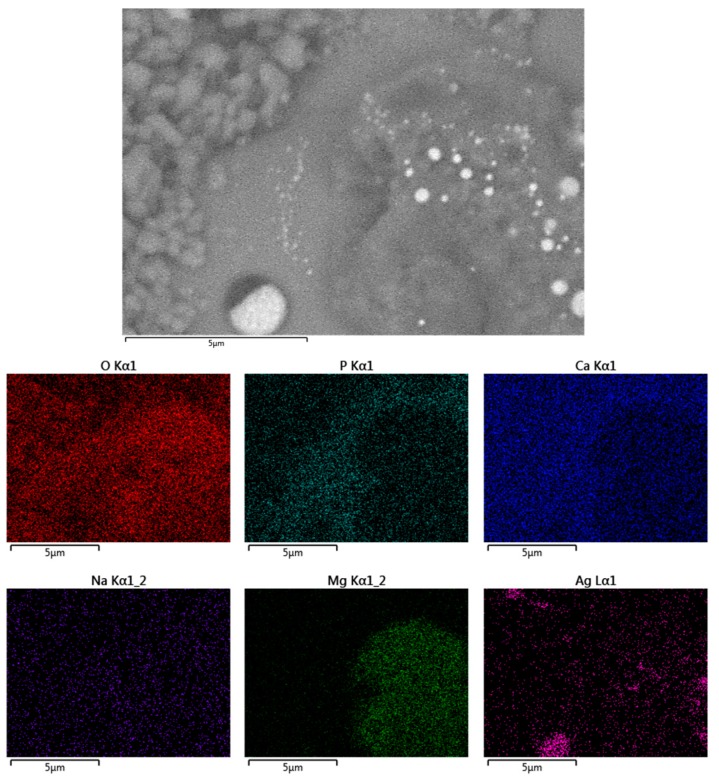
Elemental map of the HA/Ag-NPs coating.

It can be seen that the average thickness of the coating was 217.33 μm in the transverse section (Figure 5), which is above the minimum required (50 μm) to ensure a more permanent bond that guarantees implant stability with a lasting biological effect [4,26]. Elemental mapping (Figure 6) shows that Ca and P are concentrated in almost the same places. Both Ag-NPs and Na are present evenly in the coating surface.

**Figure 7:**
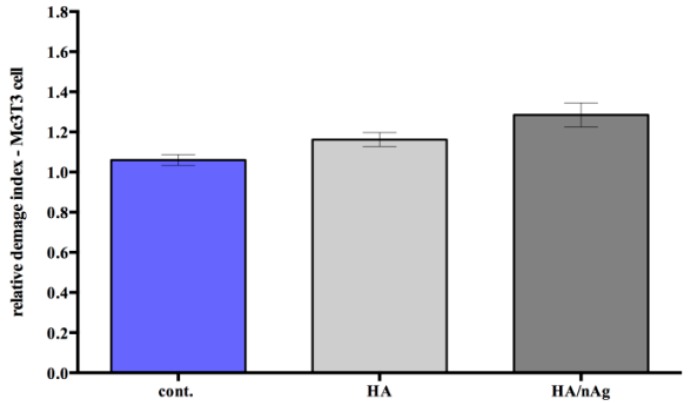
DNA damage in the osteoblast-like cells (MC3T3-E1).

**Figure 8:**
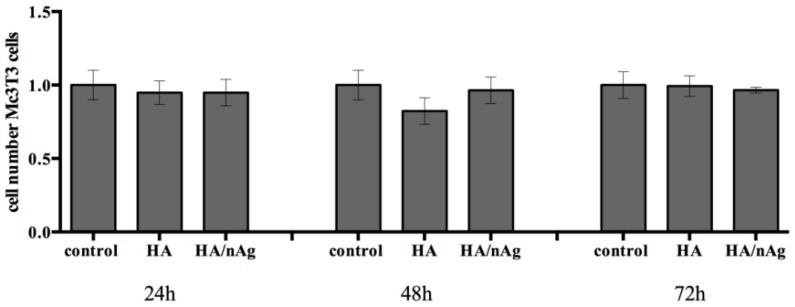
Tali image cytometry results performed on MC3T3-E1 cells to measure cell viability.

**Figure 9:**
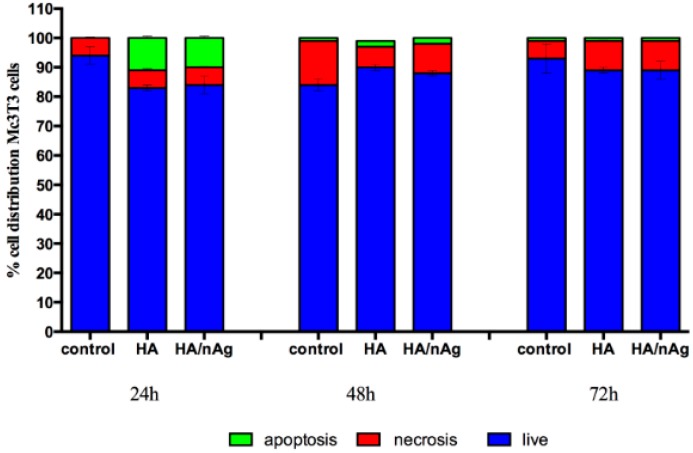
Tali image cytometry results performed with MC3T3-E1 cells.

The analysis of the DNA damage caused by the presence of the materials indicated that it does not interfere in the genetic material of the MC3T3-E1 cells (*p* < 0.05) (Figure 7). Furthermore, the cell viability analysis measured at 24, 48 and 72 h shows that the MC3T3-E1 cells on the studied coating did not grow more than controls, which indicates that the coating does not exacerbate growth, avoiding secondary effects due to an uncontrolled cell growing (Figure 8). Looking at Figure 9, we can affirm that the cells pass through an adaptation period. At the beginning, several cells died before adaptation occurred, which proves that the coating is not a harmful agent.

**Figure 10:**
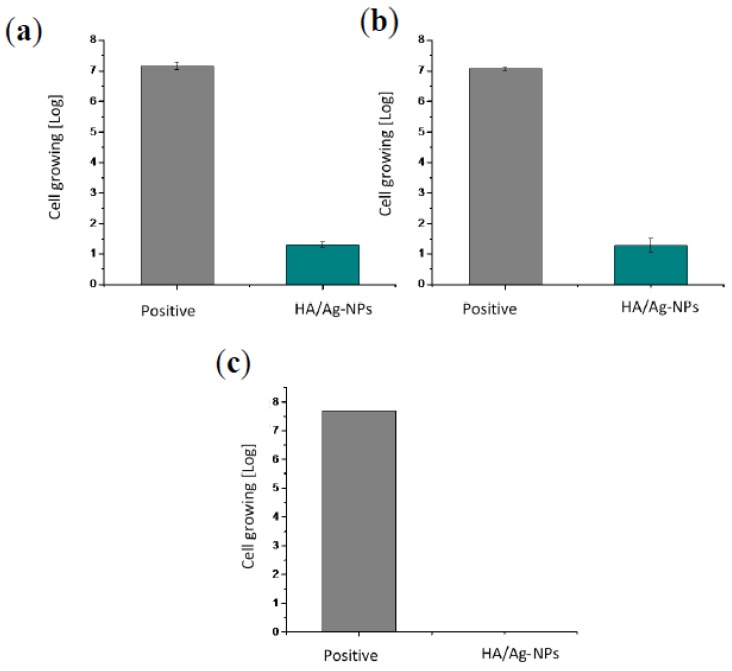
Growth of cells after 24 h of contact with a comparison of controls against HA/Ag-NPs for the following microorganisms: (**a**) *S. aureus*; (**b**) *P. aeruginosa* and (**c**) *E. coli*.

To perform the antimicrobial tests, 250,000 colony-forming units (CFU) made contact with each coating. The JIS Z 2800:2001 standard establishes that the antimicrobial value shows the difference in the logarithmic value of viable cell counts between antimicrobial products and untreated products after the inoculation and incubation of bacteria. This value is calculated as follows:

(1) $$R=\log\frac{B}{A}-\log\frac{C}{A}=\log\frac{B}{C}$$

where R is the value of antimicrobial activity; A is the average of the number of viable cells of bacteria immediately after inoculation on the untreated test piece; B is the average of the number of viable cells of bacteria on the untreated test piece after 24 h; and C is the average of the number of viable cells of bacteria on the antimicrobial test piece after 24 h [27]. Figure 10 shows that the three bacteria presented minimum activity when they were in contact with the coating for 24 h. All of the values of antimicrobial activity are above 2, which means that the coating has at least 99% antibacterial efficacy (Table 1).

## 3. Discussion

The growing interest in green chemistry methods has brought about the improvement of an eco-friendly approach for the production of Ag-NPs and biological HA. Mostly, green synthesis methods have been applied for the production of inorganic NPs using different biological systems, such as plant extracts and microorganisms [28]. Potentially, the use of coatings constituted by elements obtained by green synthesis methods will eventually minimize the problems associated with cytotoxicity in addition to increasing the bioactivity and osseointegration when the coating is installed in a host.

The current bone fragilization process involves a constant pH of 2.4 by H_3_PO_4_ addition for 8 h. This is a very important difference in comparison with other proposed bone fragilization methods, which take a longer period of time to obtain the same results. For example, Mihailescu et al. used sodium hypochlorite, which required 14 days to finish the process [29].

It is well known that the crystalline structure of HA can be modified when subjected to high temperatures (such as in an APS process) [26]. In this case, HA powder obtained from bovine bones by thermal decomposition initially presented a Ca/P ratio of 1.65. After the coating was applied by the APS process, the Ca/P ratio changed to 2.02. This indicates that there was modification in the chemical composition, probably due to the generation of other phases, such as CaO and P_2_O_5_. It has been said that the presence of CaO is not desirable because it is highly soluble and promotes coating failure. On the other hand, Nimkerdphol et al. indicated that when in contact with SBF, CaO is hydrolyzed until it is transformed to calcium carbonate (CaCO_3_) or HA [30], so a lower ratio of CaO is easily transformed to HA. The above is confirmed by SEM since the samples submerged in SBF show both superficial and chemical composition modification by energy dispersive X-ray spectroscopy (EDX). As a result, the Ca/P ratio changed with time until the value obtained was similar to HA powder (1.65).

The use of silver nanoparticles (Ag-NPs) obtained by the synthesis of nopal (*Opuntia ficus*) extracts was a good resource since nopal extract allows the Ag-NPs to have good dispersion. Furthermore, the HA/Ag-NPs mixture had good properties when sprayed via the APS process. The Ag-NPs continued being nano-sized after they were sprayed by APS. This allowed for the development of a coating with both osseointegration and biocide properties. Thus, there was no need to add more coatings [31], such as one coating of HA for osseointegration and another coating of Ag for biocide properties; or to employ two or more reagents to allow the deposition as reported by Yan et al. (Ca(NO_3_)_2_, NH_4_H_2_PO_4_ and AgNO_3_) [32].

The cytotoxicity and genotoxicity results showed that initially there was a higher cell death index due to the initial cell stress that occurs once the cells interact with the biomaterials. This behavior is commonly seen during in vitro tests. However, the biomaterial results were similar to the control results over time, indicating that the biomaterial does not interfere with cell function. The single-cell gel electrophoresis assay results indicated that the coating does not result in DNA damage. The HA/Ag-NPs performance was similar to that of the controls when studies are performed with osteoblast-like cells, indicating an excellent behavior for the intended use.

The JIS Z2801:2000 standard asserts that any material needs to perform a minimum antibacterial activity value of 2 in order to be considered as an antibacterial material. The HA/Ag-NPs coating performance was above this value for all three bacterial strains (*E. coli*, *S. aureus,* and *P. aeruginosa*), which means that it has an antibacterial efficiency of 99.99% [27].

## 4. Materials and Methods

Natural HA was obtained from bovine bones by thermal decomposition. The bovine bones were cleaned in water and boiled for 8 h at 90 °C before the bone marrow was extracted. Bones were treated for 8 h with a H_3_PO_4_ solution at a pH of 2.4, before being dried and heated in an oven for 2 h at 250 °C to induce fragility. After that, bones were grinded with a blade device until powdered.

The powder was calcined at 800 °C for 6 h and then characterized. To analyze crystallinity, a X’Pert PRO X-ray diffractometer (PANalyticial, Almelo, The Netherlands) was used, with the Cu-Kα radiation (λ = 1.5406 Å) over the Bragg angle ranging from 5° to 80°. Fourier-transform infrared spectrometry (FTIR) characterization of the powder was performed on a Spectrum GX FTIR System (Perkin Elmer, Waltham, MA, USA) with a resolution of 4 cm^−1^ in a frequency range of 4000–400 cm^−1^. The surface morphology was studied by a SU3500 Scanning Electron Microscope (HITACHI, Naka, Japan) equipped with X-Max^n^ AZtecEnergy EDS Software (OXFORD INSTRUMENTS, Abingdon, Oxfordshire, UK). The Ca/P molar ratio was determined by iCAP 6500—ICP-OES Analyzer (Thermo Fisher Scientific, Cambridge, UK). The particle size distribution (PSD) was determined in aqueous conditions using a MASTERSIZER 2000 laser diffraction particle size analyzer (Malvern, Worcestershine, UK).

Silver nanoparticles obtained by a synthesis of nopal (*Opuntia ficus*) extracts were used to give the coating antibacterial properties.

The plasma spray coating was produced using TAFA Model SG-100 plasma torch (Praxair Surface Technologies, Indianapolis, IN, USA) with the following operation parameters: 600 A, 55 psi of primary gas (Ar), 110 psi of secondary gas (He), and application distance of 50 mm [15]. The coating morphology was characterized by SEM. Coatings were exposed to a simulated body fluid (SBF) solution to analyze the apatite deposition. Ion release was analyzed by ICP-OES.

The biocompatible tests accomplished were single-cell gel electrophoresis assay and Tali image cytometry performed with osteoblast-like cells (MC3T3-E1).

The antimicrobial test was performed based on the JIS Z 2800:2001 standard. This standard mentioned that “the value of antimicrobial activity obtained by the testing methods of this standard shall not be less than 2.0 for the antimicrobial efficacy of antimicrobial products”. The value of antimicrobial activity established by the JIS Z2801:2000 standard (*R* = 2.0) is equivalent to 99.0% of antimicrobial efficacy.

## 5. Conclusions

Natural hydroxyapatite was successfully extracted from bovine bone using thermal processes, which is cleaner than industrial processes and requires less raw materials. Characterization results showed that natural hydroxyapatite has traces of ions that are necessary for cell growth, suggesting that it has better bioactivity and biocompatibility.

Applying the HA/Ag-NPs powder mix by atmospheric plasma spray allows the handling of raw material as one product. Furthermore, the presence of the nopal (*Opuntia ficus*) extract worked as a dispersant, improving the Ag-NPs dispersion on the coating surface.

HA/Ag-NPs coatings exhibited good performance in all tests, proving that 1% of silver nanoparticles is enough to inhibit bacterial growth (*S. aureus*, *E. coli* and *P. aeruginosa*), while not being enough to damage osteoblast-like cells.

## Figures and Tables

**Table 1:** Values of antimicrobial activity and efficacy of the HA/Ag-NPs coating.

	Antimicrobial Activity	Percentage of Efficacy HA/Ag-NPs
*Staphylococcus aureus*	5.6	99.99%
*Escherichia coli*	8	99.99%
*Pseudomonas aeruginosa*	5.8	99.99%
